# B Cell Subsets and Immune Checkpoint Expression in Patients with Chronic Lymphocytic Leukemia

**DOI:** 10.3390/cimb46030112

**Published:** 2024-02-23

**Authors:** Aviwe Ntsethe, Zekhethelo Alondwe Mkhwanazi, Phiwayinkosi Vusi Dludla, Bongani Brian Nkambule

**Affiliations:** 1 School of Laboratory Medicine and Medical Sciences (SLMMS), University of KwaZulu-Natal, Durban 4000, South Africa; 213512600@stu.ukzn.ac.za (A.N.); 216015946@stu.ukzn.ac.za (Z.A.M.); 2 Cochrane South Africa, South African Medical Research Council, Tygerberg 7505, South Africa; pdludla@mrc.ac.za; 3 Department of Biochemistry and Microbiology, University of Zululand, KwaDlangezwa 3886, South Africa

**Keywords:** chronic lymphocytic leukemia, immune checkpoints, B cell subsets, beta-2 microglobulin, programmed death protein 1, cytotoxic T-lymphocyte-associated protein 4

## Abstract

Chronic lymphocytic leukemia (CLL) is characterized by dysfunctional B cells. Immune checkpoint molecules such as cytotoxic T-lymphocyte-associated protein 4 (CTLA-4) and programmed death-1 (PD-1) are upregulated in patients with CLL and may correlate with prognostic markers such as beta-2 microglobulin (B2M). The aim of this study was to evaluate the levels of immune checkpoints on B cell subsets and to further correlate them with B2M levels in patients with CLL. We recruited 21 patients with CLL and 12 controls. B cell subsets and the levels of immune checkpoint expression were determined using conventional multi-color flow cytometry. Basal levels of B2M in patients with CLL were measured using an enzyme-linked immunosorbent assay. Patients with CLL had increased levels of activated B cells when compared to the control group, *p* < 0.001. The expression of PD-1 and CTLA-4 were increased on activated B cells and memory B cells, *p* < 0.05. There were no associations between B2M levels and the measured immune checkpoints on B cell subsets, after adjusting for sex and age. In our cohort, the patients with CLL expressed elevated levels of PD-1 and CTLA-4 immune checkpoints on activated and memory B cell subsets. However, there was no correlation between these immune checkpoint expressions and B2M levels.

## 1. Introduction 

Chronic lymphocytic leukemia (CLL) is the most common type of leukemia among adults, with a global prevalence of about 3.5 cases per 100,000 people [1]. In high-income countries, CLL accounts for more than a third of all leukemia cases [1]. Notably, low-to-middle-income countries have a five- to ten-fold lower age-adjusted incidence rate of CLL compared to high-income countries [2]. CLL is a lymphoproliferative disorder that is characterized by functionally incompetent B cells with a distinct immunophenotype [3]. However, there are divergent findings regarding the predictive significance of B cell subsets in leukemia [4].

Regulatory B cells (Bregs) modulate T cell-driven anti-tumor immunity, promoting the expression of forkhead box protein 3 (FoxP3+) in regulatory T cells (T-regs), which dampens the innate and adaptive antitumor immune response [4,5]. These immunosuppressive mechanisms involve Bregs, which acquire inhibitory ligands and signal transducer and activator of transcription 3 (STAT3) phosphorylation, and the induction of interleukin 10 (IL-10) and transforming growth factor-β (TGF-β) [4,5]. The expression of inhibitory molecules such as programmed death-1 (PD-1) or programmed death-ligand 1 (PD-L1) inhibits the anti-tumor function of Bregs [4]. In patients with CLL, immune checkpoints such as T-cell immunoglobulin-3 (TIM-3), cytotoxic T-lymphocyte-associated protein 4 (CTLA-4), and programmed death-1 (PD-1) are upregulated [6,7]. The use of immune checkpoint inhibitors (ICI) such as PD-1 antagonists has fundamentally altered therapeutic approaches in patients with CLL [8]. Targeting PD-1 has been shown to improve the overall survival of patients with CLL while also reducing adverse effects [8]. In patients with relapsed CLL, zanubrutinib, a Bruton’s tyrosine kinase inhibitor, is associated with fewer cardiac adverse events and improved progression-free survival [9]. 

There has been a significant transformation in the management of patients with CLL. Advances in prognostic markers and enhanced predictive capabilities for clinical outcomes have collectively made a vital impact on the management of CLL. The important adverse prognostic markers include immunoglobulin heavy-chain variable region gene (IGHV) mutation status, zeta-chain-associated protein kinase-70 (ZAP-70) and CD38 expression, and beta 2 microglobulin (B2M) levels [10]. The levels of B2M are considered an independent marker for poor prognosis in patients with CLL [11]. The serum levels of B2M are elevated in patients with CLL who are at an advanced stage of the disease, and these levels decrease post-treatment [12]. The primary aim of this study was to determine the levels of immune checkpoint expression on peripheral B cell subsets in patients with CLL and to further correlate these immune checkpoints with B2M levels in patients with CLL. 

## 2. Methods and Materials

### 2.1. Patient Recruitment

Patients and healthy control participants were recruited between July 2019 and May 2022 from King Edward VIII Hospital, a tertiary healthcare facility located in Durban, KwaZulu-Natal, South Africa. The study was conducted in accordance with the Declaration of Helsinki, and ethical approval was obtained from the University of KwaZulu-Natal Biomedical Research Ethics Committee (BE456/18), South Africa. Written informed consent was obtained from all the study participants. In this study, we excluded patients with CLL who were on treatment, and only untreated patients were included along with age-matched healthy controls with no clinical signs of infection. The ethnicity of the participants was self-reported.

### 2.2. Sample Collection

Peripheral blood was collected from consenting participants by venipuncture into 6 mL ethylenediaminetetraacetic acid (EDTA) tubes (BD Bioscience, San Jose, CA, USA). The samples were transported at room temperature from the hospital to the laboratory. 

### 2.3. Hematological Analysis

Hematological parameters including white blood cell count, hemoglobin, and platelet count were measured using an automated Coulter AcT Diff hematology analyzer (Beckman Coulter Inc., Brea, CA, USA) within 1–2 h of the peripheral blood collection. 

### 2.4. Isolation of Peripheral Blood Mononuclear Cells (PBMCs)

The peripheral blood mononuclear cells (PBMCs) were isolated from the whole blood samples by the density gradient centrifugation method using the Ficoll-Paque PLUS (Amersham, Biosciences, Uppsala, Sweden), as previously described [13]. Briefly, 3 mL of Ficoll-Paque PLUS was aliquoted into a 15 mL centrifuge tube (Sigma-Aldrich, St. Louis, MI, USA), 4 mL of whole blood was carefully layered on the Ficoll-Paque PLUS gradient, and the samples were centrifuged at 400× *g* for 40 min at 20 °C. The PBMC layer was then collected and stored at −80 °C.

### 2.5. T Cell Depletion and B Cell Isolation from Peripheral Blood Mononuclear Cells

To enrich the B cell population from the collected PBMCs, we performed T cell depletion and positively selected B cells using the BD IMag isolation system (BD Bioscience, San Jose, CA, USA). Briefly, 50 µL of PBMCs were incubated with 5 µL of biotinylated human T lymphocyte enrichment cocktail (BD Biosciences, San Jose, CA, USA) for 15 min at room temperature. After the incubation, 50 µL of streptavidin particles were added to the T cell-depleted PBMC samples and incubated for 30 min at room temperature. The samples were then reconstituted in 1 mL of 3.2% sodium citrated buffer, and the samples were placed on the BD Imag for 8 min. The isolated B cells were reconstituted in 100 µL of PBS and stored at −80 °C.

### 2.6. Measurements of B Cell Subsets

**Figure 1:**
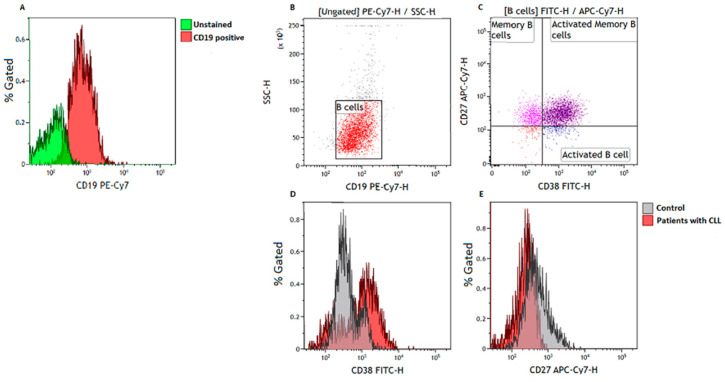
Gating strategy. (**A**) histogram illustrating the separation of unstained cells and CD19-positive cells; (**B**) B cells were gated based on side scatter (SSC) and CD19 expression; (**C**) illustrates the gating of activated B cells defined as CD19^+^CD38^+^CD27^−^, memory B cells defined as CD19^+^CD27^+^ events, and activated memory B cells defined as CD19^+^CD27^+^CD38^+^ events, respectively. Histograms demonstrate the levels of activated B cells (**D**) and memory B cells (**E**) in the control group and patients with CLL.

To quantify the B cell subsets, we made use of a six-color flow cytometry panel consisting of CD38-FITC, CD152-PE, CD273-APC, CD19-PE/Cy7, CD27-APC/Cy7, and CD279-BV421 (BioLegend, San Diego, CA, USA). The approach of immunophenotyping the overall B cell population is important in understanding the overall heterogeneity of B cell responses. According to Wei et al. [14], diversity in B cell responses plays a crucial role in shaping the effectiveness of immune responses in B cell malignancies. We acquired at least 5000 B cells (CD19^+^ events) (Figure 1A) and defined memory B cells as CD19^+^CD27^+^ events [15,16] (Figure 1B), activated B cells as CD19^+^CD27^−^CD38^+^ events, and activated memory B cells [16] as CD19^+^CD27^+^CD38^+^ events (Figure 1C). Details can be found in the Minimum Information about a Flow Cytometry Experiment (MIFlowCyt) document (Supplementary Table S2). 

### 2.7. Measurements of Immune Checkpoint Levels on B Cell Subsets

To determine the levels of immune checkpoint expression on B cell subsets, we measured the expression of CD279 (PD-1), CD273 (PD-L2), and CD152 (CTLA-4) on B_MEM_ and activated B cells. A total of 5000 CD19^+^ events were acquired at a medium flow rate. All the data were acquired using the BD FACSCanto II flow cytometer (BD Biosciences, San Diego, CA, USA) and analyzed using the Kaluza version 1.2 (Beckman Coulter Inc., Brea, CA, USA).

### 2.8. Measurements of Serum Soluble Beta-2 Microglobulin (B2M) Levels 

To measure the plasma levels of B2M, a prognostic marker in patients with CLL [17], we made use of a beta-2 microglobulin human enzyme-linked immunosorbent assay kit (ThermoFisher Scientific, Waltham, MA, USA), according to the manufacturer’s instruction.

### 2.9. Sample Size Estimation

We calculated the minimum sample size of patients needed to detect a large effect size (d) of 1.14 in the expression of immune checkpoints, namely PD-1 and PD-L1 at 80% power and alpha (α) of 0.05 using GPower 3.1.94 software (Universität, Düsseldorf, Germany). To detect a large effect size between two independent means using an unpaired *t*-test, we required a minimum of twenty-one patients with CLL (*n* = 21) and twelve controls (*n* = 12).

### 2.10. Statistical Analysis

All statistical analysis was performed using GraphPad Prism version 8 software, (GraphPad Software Inc., San Diego, CA, USA) and [18]. All non-parametric data were log-transformed prior to statistical analysis and reported as the mean and standard deviation. An unpaired Student’s *t*-test was performed to compare parametric data between the two groups. A repeated measures one-way ANOVA was used to compare parametric multiple data of the same group, with Dunnett’s as a post hoc test for multiple comparisons. To correct for multiple comparisons, a Bonferroni-corrected critical *p*-value of < 0.0167 was considered statistically significant. 

## 3. Results

### 3.1. Patient Characteristics and Hematological Parameters 

This study comprised 21 patients with CLL and 12 healthy controls. The mean age of the patients with CLL was 62.33 ± 13.31 years and 56.58 ± 15.67 years for the controls. The included study participants were from different ethnic groups, which comprised African (*n* = 30), European (*n* = 2), and Indian (*n* = 1). The study included 39.39% females and 60.61% males.

Based on the treatment initiation criteria, sixteen patients were on a “watch and wait” approach, and five had a treatment indication at the time of sample collection [19]. In the patients with CLL, the white blood cell count was significantly increased (130.4 × 10^3^ ± 29.71) compared to the controls (5.26 × 10^3^ ± 1.38), *p* = 0.0005) (Table 1), whereas the red blood cell count and hemoglobin were significantly reduced in patients with CLL compared to age-matched control patients (*p* < 0.0001). There were no statistically significant differences in the platelet count in patients with CLL when compared to the control group (*p* = 0.1831).

### 3.2. Increased Levels of Activated B Cells in Patients with CLL

**Figure 2:**
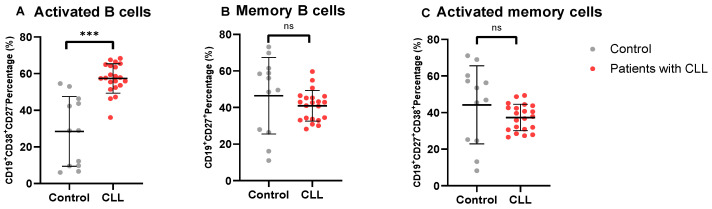
B cell subsets in patients with CLL. (**A**–**C**) depict the levels of activated B cells, memory B cells, and activated memory B cells in patients with CLL compared to healthy controls. The data are presented as the mean ± standard deviation (SD). *** shows the level of significance between groups, ns (not significant).

We evaluated the levels of B cell subsets in patients with CLL (Figure 2). Notably, patients with CLL had significantly increased levels of activated B cells (57.39 ± 8.001) compared to the control group (28.47 ± 19.01; *p* = 0.0002). 

Furthermore, the expression of memory B cells in patients with CLL (40.95 ± 8.353) was found to be statistically comparable to that in the control group (46.45 ± 20.90; *p* = 0.3984), as was the expression of activated memory B cells expressing (37.31 ± 7.191) in patients with CLL compared to the control group (44.25 ± 21.34; *p* = 0.2956). 

### 3.3. Elevated Levels of PD-1 Expression on Activated B Cells and Memory B Cells in Patients with CLL

**Figure 3:**
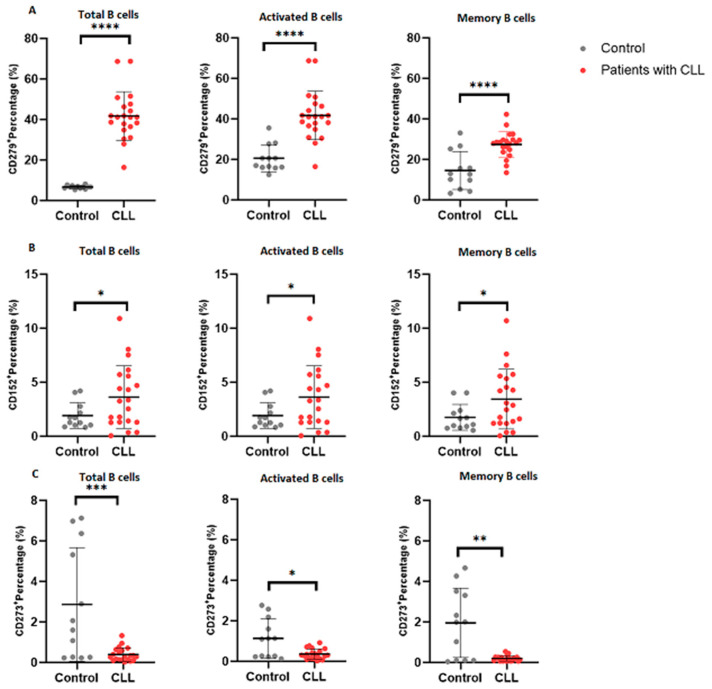
Immune checkpoint expression on B cell subsets. This figure depicts the expression levels of (**A**) PD-1, (**B**) CTLA-4, and (**C**) PD-L2 on total B cells, activated B cells, and memory B cells, respectively. The data are presented as the mean ± standard deviation (SD). *, **, *** and **** shows the level of significance between groups.

Significantly elevated PD-1 expression was observed on B cells from patients with CLL (40.20 ± 6.601) compared to the control group (6.690 ± 0.8160; *p* < 0.0001) (Figure 3A). Likewise, PD-1 expression was increased on activated B cells in patients with CLL (5.325 ± 0.4397) relative to the control group (4.305 ± 0.4317; *p* < 0.0001). Moreover, a notable increase in PD-1 expression was noted on memory B cells in patients with CLL (27.44 ± 6.358) compared to the control group (14.59 ± 9.395; *p* < 0.0001). 

### 3.4. Elevated Levels of CTLA-4 on Activated and Memory B Cells in Patients with CLL

A significant increase in CTLA-4 expression was detected on B cells in patients with CLL (3.630 ± 2.897) compared to the control group (1.919 ± 1.184; *p* = 0.0241) (Figure 3B). Similarly, CTLA-4 expression was elevated on activated B cells in patients with CLL (3.631 ± 2.896) relative to the control group (1.919 ± 1.184; *p* = 0.0240). Notably, CTLA-4 expression on memory B cells in patients with CLL (3.439 ± 2.767) was higher compared to the control group (1.755 ± 1.205; *p* = 0.0221). 

### 3.5. Decreased Levels of PD-L2 Expression on Activated and Memory B Cells in Patients with CLL

Remarkably, PD-L2 expression was significantly decreased on B cells in patients with CLL (0.3967 ± 0.3343) compared to the control group (2.870 ± 2.800; *p* = 0.0003) (Figure 3C). A similar decrease in PD-L2 expression was observed on activated B cells in patients with CLL (0.3624 ± 0.2514) compared to the control group (1.137 ± 0.9655; *p* = 0.0186). Additionally, PD-L2 expression on memory B cells in patients with CLL (0.2005 ± 0.1297) was reduced compared to the control group (1.956 ± 1.706; *p* = 0.0044). 

### 3.6. Analysis of Immune Checkpoint (PD-1 and CTLA-4) Expression among B Cell Subsets in Patients with CLL

**Figure 4:**
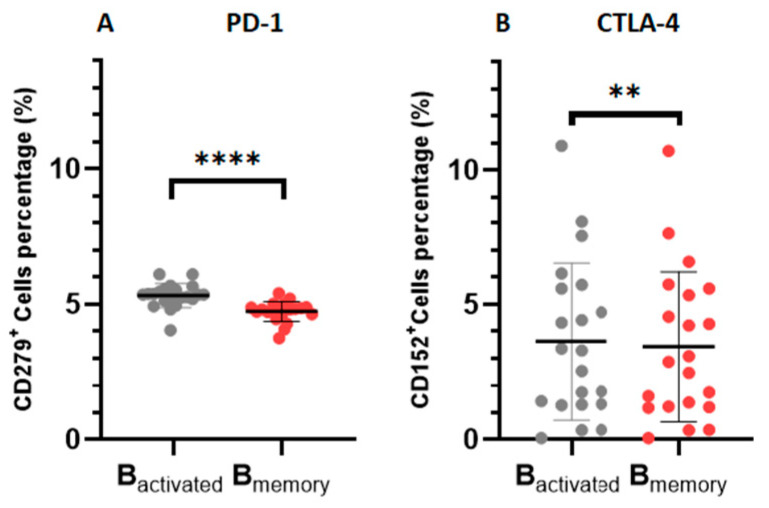
Immune checkpoint expression on B cell subsets. (**A**) illustrates the expression of PD-1 on activated (CD19^+^CD38^+^ cells) and memory B cells (CD19^+^CD27^+^ cells). (**B**) illustrates the levels of CTLA-4 expression on activated B cells and memory B cells. All data are presented as the mean ± standard deviation (SD). ** and **** shows the level of significance between groups.

In order to elucidate which B cell subsets were associated with heightened immune checkpoint expression, we conducted a comparative assessment of immune checkpoint expression within various B cell subsets in patients with CLL (Figure 4). PD-1 levels were significantly elevated on activated B cells when compared to memory B cells (0.5877 ± 0.04654, *p* < 0.0001). Similarly, CTLA-4 expression activity was found to be elevated when compared to memory B cells (0.1924 ± 0.04848, *p* = 0.0039). 

### 3.7. β2 Microglobulin Levels Are Not Associated with the Expression of PD-1 or CTLA-4 on B Cell Subsets

The levels of B2M are independently associated with disease progression in patients with CLL [17]. In our multivariable regression model, there was no association between B2M levels and PD-1 on B cells (β = 0.008, SE = 0.011, *p* = 0.524), PD-1 on activated B cells (β = 0.008, SE = 0.011, *p* = 0.525), PD-1 on memory B cells (β = 0.0008, SE = 0.004, *p* = 0.905), CTLA-4 on B cells (β = 0.004, SE = 0.002, *p* = 0.426), CTLA-4 on activated B cells (β = 0.004, SE = 0.002, *p* = 0.419), or CTLA-4 on memory B cells (β = 0.004, SE = 0.002, *p* = 0.419) in patients with CLL (Supplementary Table S1).

## 4. Discussion

The primary aim of this study was to determine the levels of immune checkpoint expression on the peripheral B cell subsets in patients with CLL and to further correlate these immune checkpoints with B2M levels, a confirmed independent prognostic marker in patients with CLL [17]. In this study, we determined the baseline expression levels of B cell subsets in patients with CLL. The patients with CLL had a higher activated B cell fraction (CD38^+^ B cells), which has already been described in patients with CLL [20]. In our study, the activated B cells had a CD38 phenotype, which is one of the prognostic markers for patients with CLL [21,22]. Notably, the activated B cell profile is not restricted to peripheral circulation [23], and the expression of CD38 is associated with an increased proliferation index [24,25]. The increased levels of CD38^+^ activated B cells in patients with CLL may indicate patients with a poor survival rate and response to therapy [20,21,22]. Moreover, in our study, patients with CLL expressed higher CTLA-4 and PD-1 immune checkpoint proteins on activated B cell subsets. The levels of PD-1 and CTLA-4 were higher on activated B cells when compared to the other B cell subsets.

The relevance of PD-1 expression on CD4^+^ T cells has been well described in patients with CLL at an advanced stage (Rai III/IV) [26]. To date, the exhaustion levels of B cell subsets in patients with CLL have not been investigated. Immune system dysfunction is a hallmark of CLL, which primarily affects humoral immunity and is characterized by increased susceptibility to autoimmune disorders and secondary malignancies [27,28]. 

Immune checkpoint proteins such as PD-1, PD-L1, PD-L2, and CTLA-4 are expressed on activated B cells [29,30]. The function of these immune checkpoints on B cells has not been fully evaluated. However, the binding of PD-L1 or PD-L2 to PD-1 receptors on T cells is known to induce phosphorylation of ITIM (Immunoreceptor Tyrosine-Based Inhibitory Motif) and ITSM (Immunoreceptor Tyrosine-Based Switch Motif) within the PD-1 receptor [31]. This phosphorylation leads to the recruitment of phosphatases such as Src Homology 2 Domain-Containing Protein Tyrosine Phosphatase 2 and Src Homology 2 Domain-Containing Protein Tyrosine Phosphatase 1 [31,32]. Consequently, these phosphatases dephosphorylate key signaling molecules downstream of the T-cell receptor (TCR), such as PI3K (Phosphoinositide 3-Kinase) and AKT (Protein Kinase B) [33,34,35]. The overall effect of PD-1 binding is to inhibit the proliferation of B cells, leading to immune suppression and tumor immune evasion [36]. 

In our study, we further observed a significant increase in PD-1 levels on B cells and memory B cells among patients with CLL. Furthermore, PD-L2 expression was lower across the B cell subsets in patients with CLL. These findings underscore the complex and dynamic interplay between immune checkpoint molecules and distinct B cell populations within the CLL microenvironment. PD-1 function in T cells is widely studied and is associated with T cell exhaustion in patients with CLL [37]. However, its role on B cells is not known. In our study, we showed that PD-1 is expressed strongly on activated B cells. Thibult et al. [36] showed that PD-1 and its ligands (PD-L1 and PD-L2) are key regulatory proteins of B cell activation and entry into the germinal center. Moreover, the study demonstrated that PD-1 is the inhibitor of the toll-like receptor (TLR) ligand-mediated activation of B cells. The increased expression of PD-1 may inhibit B cell differentiation into antibody secreting cells. PD-1 has a higher affinity for PD-L1 than PD-L2 [36,38]; this may explain the significant decrease in PD-L2 expression in patients with CLL. 

In this study, we observed a significant increase in CTLA-4 levels on B cells and memory B cells among patients with CLL. CTLA-4 suppresses humoral response to T cell-dependent and independent antigens [39]. In addition, elevated CTLA-4 expression on B cells is associated with disease progression in patients with CLL [40]. Moreover, the elevated expression of this immune checkpoint in peripheral blood inhibits B cell activation and proliferation [41]. Consistent with this study, our study found increased levels of CTLA-4 expression in B cell subsets. Therefore, the use of immune checkpoint inhibitors targeting CTLA-4 in B cells may be beneficial in patients with CLL. 

In this study, we determined the relationship between immune checkpoints on B cell subsets and B2M. There was no correlation between B2M levels and the expression of immune checkpoint molecules on B cell subsets. The elevated B2M levels are associated with an advanced CLL stage [42]. In CLL, B2M levels can be elevated due to increased turnover of CLL cells or other factors. The elevated B2M is associated with advanced CLL stages [42]. While immune checkpoints are dysregulated in CLL, B2M levels do not directly correlate with the expression of these checkpoints in B cell subsets. This suggests that the expression of these immune checkpoints in B cell subsets does not directly influence B2M levels. However, on the CLL international prognostic index (IPI), we only investigated correlation with β2-microglobulin.

## 5. Conclusions

In our cohort, the patients with CLL exhibited increased levels of PD-1 and CTLA-4 on B cell subsets. However, the expression of these immune checkpoint molecules on B cell subsets did not correlate with B2M levels.

## Figures and Tables

**Table 1:** The baseline characteristics and hematological parameters of the participants.

	Control (*n* = 12)	Patients with CLL (*n* = 21)	*p*-Value
Age (Years)	56.58 ± 15.67	62.33 ± 13.31	0.2714
Male, *n* (%)	58.33	61.9	
Female, *n* (%)	41.67	38.1	
White blood cell count (10^3^ µL)	5.26 ± 1.38	130.4 ± 29.71	0.0005
Red blood cell (10^6^ µL)	4.74 ± 0.94	2.10 ± 0.84	<0.0001
Hemoglobin (g/dL)	14.13 ± 3.81	8.19 ± 2.30	<0.0001
Platelets (10^3^ µL)	210.4 ± 73.14	157.5 ± 141.9	0.1831

## Data Availability

The data are contained within the article and Supplementary Materials. The data generated in this study are available upon request from the corresponding author.

## Supplementary Materials

The following supporting information can be downloaded at: https://www.mdpi.com/article/10.3390/cimb46030112/s1, Table S1: Age, sex adjusted multivariable regression analysis of immune checkpoints on B cell subsets with B2M; Table S2: Minimum Information about a Flow Cytometry Experiment (MIFlowCyt).
