# Supplementary material for: B Cell Subsets and Immune Checkpoint Expression in Patients with Chronic Lymphocytic Leukemia
Source: Curr Issues Mol Biol. 2024 Feb 23;46(3):1731–40. doi: 10.3390/cimb46030112 (PMC10969689; doi:10.3390/cimb46030112)
Supplement: Supplementary file 1 [file cimb-46-00112-s001.zip › cimb-2842722-supplementary.pdf]

Supplementary Materials

Table S1. Age, sex adjusted multivariable regression analysis of immune checkpoints on B cell subsets with B2M.

	**B2M**		**Gender**		**Age (years)**	
	**Coefficient Estimate (SE)**	***p*-Value**	**Coefficient Estimate (SE)**	***p*-Value**	**Coefficient Estimate (SE)**	***p*-Value**
PD-1 on B cells	0.008 (0.011)	0.454	−2.811 (7.206)	0.701	−0.179 (0.276)	0.524
PD-1 on activated B cells	0.008 (0.011)	0.458	−2.825 (7.192)	0.699	−0.179 (0.275)	0.525
PD-1 on memory B cells	0.0008 (0.006)	0.897	−0.929 (3.900)	0.815	−0.018 (0.149)	0.905
CTLA-4 on B cells	0.004 (0.002)	0.098	−0.171 (1.631)	0.918	−0.051 (0.062)	0.426
CTLA-4 on activated B cells	0.004 (0.002)	0.086	−0.056 (1.548)	0.972	−0.049 (0.059)	0.419
CTLA-4 on memory B cells	0.004 (0.002)	0.086	−0.056 (1.548)	0.972	−0.049 (0.059)	0.419

Table S2. Minimum Information about a Flow Cytometry Experiment (MIFlowCyt).

**Instrument Manufacturer**	**BD Biosciences**
Instrument model	FACSCanto™ II
Instrument configuration and settings	Detector voltages were set using cytometer setup and tracking (CST) beads (BD Bioscience, Franklin Lakes, NJ, USA), 5000 B cells ($CD19^+$ events) were acquired on stained samples a medium flow rate on an FACS Canto II, BD Biosciences, equipped with three lasers, a blue (488-nm), a red (633-nm) and a violet (405-nm).
Compensation description	BD™ Compbead compensation particles (BD Bioscience, Franklin Lakes, NJ, USA) were used to determine the levels of spectral overlap and perform color compensation.
Gate description	A gate was applied manually using Kaluza version 1.2 (Beckman coulter, Inc., Brea, CA, USA). B cells were defined as $CD19^+$ events, memory B cells as $CD19^+CD27^+$ events, activated B cells as $CD19^+CD27^-CD38^+$ events and activated memory B cells as $CD19^+CD27^+CD38^+$ events. Expression of CD279 (PD-1), CD273 (PD-L2) and CD152 (CTLA-4) on total B cells, activated B cells and memory B cells.
